# Functioning and Safety of the Non-Invasive Corneal Esthesiometer Brill: A Multicenter Study

**DOI:** 10.3390/diagnostics15172208

**Published:** 2025-08-30

**Authors:** Concepción Renedo Laguna, Carmen Gómez Martín, Javier Lozano-Sanroma, José Manuel Benítez del Castillo, Jesús Montero Iruzubieta, Salvador García Delpech, Jesús Merayo-Lloves

**Affiliations:** 1 Instituto Universitario Fernández Vega, 33012 Oviedo, Spain; 2 Brill Engines, S.L., 08022 Barcelona, Spain; 3 Optometry Deparment, Universidad de Oviedo, 33003 Oviedo, Spain; 4 Clínica Rementería, 28010 Madrid, Spain; 5 Facultad de Medicina, Ophthalmology Deparment, Universidad Complutense de Madrid, 28040 Madrid, Spain; 6 Clínica Cartujavisión, 41092 Sevilla, Spain; 7 Clínica Aiken, 46004 Valencia, Spain

**Keywords:** esthesiometer, corneal sensitivity, cornea, sensitivity loss, ocular conditions

## Abstract

**Background/Objectives**: Corneal sensitivity can be decreased by several ocular conditions, including dry eye and refractive surgery, which can lead to ocular epithelial lesions. This decrease can be detected by esthesiometry. The main objective of this study was to evaluate the performance, safety, and efficacy of the Corneal Esthesiometer Brill in healthy subjects without ocular pathologies. **Methods**: A controlled, randomized, prospective, multicenter pilot clinical study was conducted in adult patients with healthy eyes. Corneal sensitivity measurements were made three times for one eye randomly selected to obtain the corneal sensitivity reference ranges. Additionally, one more measurement was taken after the application of a topical anesthetic. An intra- and inter-observer analysis was performed to assess user dependence, and the last measurement was taken after ocular topical anesthesia to evaluate the device’s sensitivity in detecting corneal sensitivity loss. **Results**: Ninety-one volunteers were included with a mean age of 25 (SD 3.46, range 18–30), and fifty-eight (63.7%) were female. Corneal sensitivity reference levels ranged from level 2 (3–4 mbar) to level 3 (4–5 mbar). Intra- and inter-observer measurement differences on the same subject without anesthesia were not statistically significant. Corneal pressure before and after local ocular anesthesia had statistically significant differences (*p* < 0.0001). **Conclusions**: The Corneal Esthesiometer Brill yielded consistent and reproducible measurements in young volunteers with healthy eyes, enabling objective, observer-independent use and facilitating the detection of significant loss of sensitivity.

## 1. Introduction

The cornea is one of the body’s most densely innervated and sensitive tissues [1]. Corneal nerves protect the cornea, stimulating the expression of corneal collagen and the epithelial cell function by regulating tear production and eyelid closure [2,3]. Ocular conditions such as dry eyes [4], conjunctivitis, diabetic corneal neuropathy [5], glaucoma, uveitis, herpes zoster [6], or keratitis, and also, aging, contact lens wear, refractive surgery, keratoplasty [7], or trauma [5], can affect corneal sensitivity producing a decrease in sensitivity by a corneal decreased nerve density [8,9], epithelial defects, and even, blindness [10]. The loss of corneal sensitivity can result in a reduction in blinking and tearing reflexes, which may contribute to the development of lesions in the corneal epithelium [11]. In these cases, the epithelial defects and corneal epithelium injuries can persist due to a deficiency in the healing process [12]. For example, patients with diabetic corneal neuropathy often suffer from ocular irritation or pain [13], but in many cases, are asymptomatic due to a corneal hypoesthesia [14]. Therefore, it is crucial to have sensitive tools to detect an early reduction in ocular sensitivity [2].

Corneal sensitivity can be assessed both qualitatively and quantitatively [15]. Qualitative evaluation can be performed using a cotton swab [16]. This method records the sensation in each corneal area depending on whether it is “normal,” “reduced,” or “non-existent.” Quantitative measurement is commonly measured using an esthesiometer [15]. Considered the gold standard, the most used is the Cochet-Bonnet esthesiometer (CBE). This device uses a nylon filament of adjustable length to test mechanical sensitivity (<5 mm suggests hyposensitivity, 6 mm suggests complete sensitivity) [17]. Currently, other esthesiometers are available, such as Belmonte esthesiometer, a non-contact esthesiometer using puffs of air at different temperatures, pressure, or CO_2_ concentrations [18]; the Swiss Liquid Jet Esthesiometer that delivers fine droplets on the ocular surface with a pulsed stimulus mode [19]; or the Corneal Esthesiometer Brill (CEB) [20], portable non-contact esthesiometer which provides up to five pulses of air (ambient air) without the need to incorporate pumps with other gases with a constant pulse that lasts up to 0.5 s on the surface of the assessed eye [21]. The principal difference between contact and non-contact esthesiometers is the disruption of the epithelial surface produced by contact and the stimulation of corneal nociceptors. Additionally, contact esthesiometers can cause apprehension, which affects the measurements and makes it impossible to mask the filament [22,23].

Because the CEB is a relatively new non-contact device for measuring corneal sensitivity, few studies on its clinical performance are currently available. No multicenter studies have been conducted to validate its reproducibility, safety, or ability to detect changes in sensitivity in healthy populations. The main objective of this study, conducted in four ophthalmological centers, was to assess the performance, safety, and efficacy of CEB in healthy subjects without ocular pathologies.

## 2. Materials and Methods

### 2.1. Study Design and Subjects

A non-controlled, randomized, prospective, multicenter pilot clinical study was carried out at the Instituto Oftalmológico Fernández-Vega (Oviedo, Spain), Clínica Rementería (Madrid, Spain), Clínica Cartuja Visión (Sevilla, Spain), and Clínica Aiken (Valencia, Spain) from March 2022 to December 2022. Patients included were healthy adults with healthy eyes. Ocular sensitivity was evaluated with the CEB (Brill Engines, Barcelona, Spain), a device registered in Europe as a Class IIa Medical Device (CE mark) and currently under registration with the Food and Drug Administration (FDA).

The protocol was approved by the “Research Ethics Committee of the Principality of Asturias,” and the study was carried out following the principles established in the revised version of the Declaration of Helsinki and Good Clinical Practices. All patients signed informed consent before any procedure.

Eligible participants included healthy volunteers between 18 and 30 years of both sexes with dark eyes, no previously diagnosed ocular pathology, normal ocular surface disease index (OSDI) with a score < 13, correct slit-lamp biomicroscopy, and without ophthalmic or systemic treatments, which may alter the corneal sensitivity. Patients excluded were those with dry eye syndrome (OSDI score > 13 or incorrect slit-lamp biomicroscopy); acute ocular disorders or diseases such as infectious conjunctivitis, ocular allergy, inflammation of the anterior chamber, and anterior segment dysgenesis; ocular chronic disorders such as disorders of the tear drainage system, glaucoma, and meibomian gland dysfunction; autoimmune diseases that require treatment; systemic, neurological, and/or dermatological diseases with ocular involvement; eye surgery/blepharoplasty; treatments with topical medication or systemic medication involving tear production in the last three months; or patients using contact lenses.

### 2.2. Primary and Secondary Endpoints

The primary efficacy endpoint was determining the corneal sensitivity reference ranges in young, healthy volunteers without ocular pathologies. Secondary endpoints included evaluating the CEB efficacy and reproducibility in healthy subjects, assessing intra- and inter-observer variability when performing esthesiometry on the same subject without anesthesia, and evaluating CEB sensitivity by determining corneal pressure differences in healthy volunteers before and after ocular local anesthesia.

### 2.3. Variables Assessed

Corneal sensitivity reference ranges were assessed by applying a series of air pulse pressures to the cornea of a healthy volunteer, starting at the lowest pressure level and increasing the pressure until the subject felt the air pulse.

### 2.4. Study Visits and Procedures

**Figure 1:**
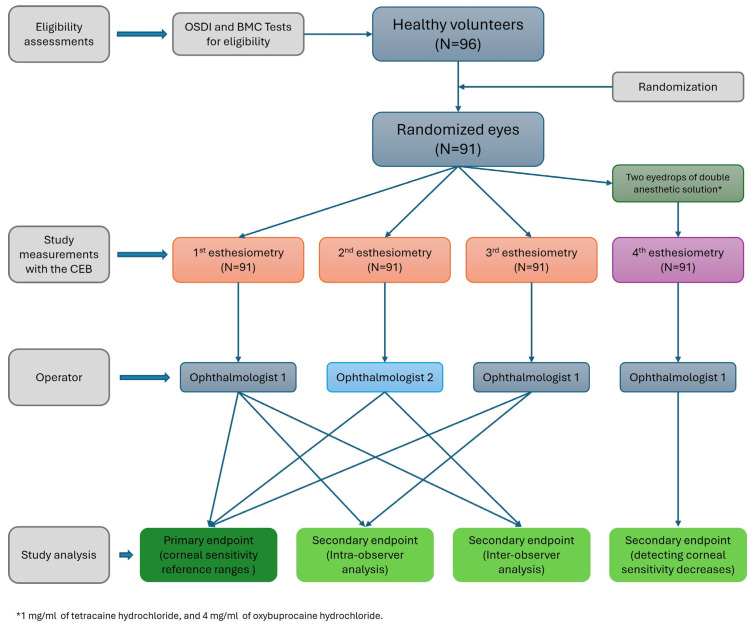
The study flowchart. Abbreviations: CEB, Corneal Esthesiometer Brill.

The study lasted twelve months and one day for each patient. All procedures used to assess efficacy were standard. Before starting the study, a training session was conducted to ensure the quality of the data, and the sponsor monitored both the investigators and the study staff (Figure 1).

To confirm the volunteer eligibility, the ocular surface of both eyes was checked using first the OSDI test, a simple test created to establish the severity and classification of the dry eye disease according to its symptoms [24], and subsequently, using a biomicroscope in the slit lamp was carried out according to the manufacturer’s instructions [25].

### 2.5. Corneal Sensitivity Assessment with the CEB

**Figure 2:**
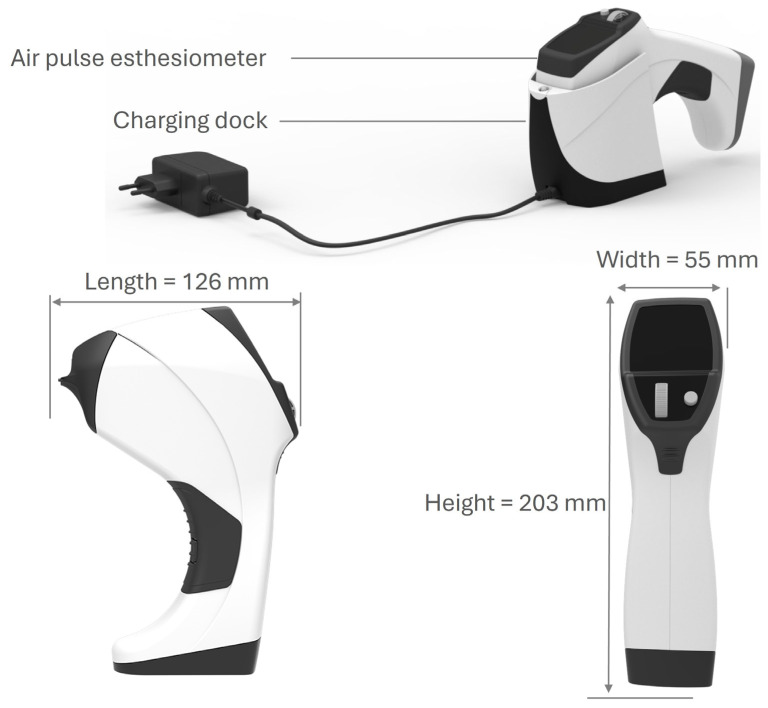
The CEB device: external design and dimensions.

In all participants, the sensory function of the ocular surface was measured through corneal sensitivity using esthesiometry. The assessment was performed with the CEB in the central part of the cornea of one eye of each participant, previously randomized, four times on the same day. The CEB has five pressure levels (levels 1 to 5) and was designed to be placed at a certain distance between the esthesiometer’s air output nozzle and the patient’s corneal surface (Figure 2). This distance corresponds to the point where two light-emitting diodes (LEDs) converge on the same spot on the patient’s cornea and is used to guide the air jet. The proper distance is approximately 4 mm, which allows for very low dispersion. The air pressure, with which it reaches the ocular surface, varies very little with the output pressure. Furthermore, at this distance, the air contact area on the corneal surface can be as small or as similar as possible to the output area of the esthesiometer’s nozzle.

For each assessment, two values were obtained: (1) the pressure level at which the air jet was delivered, and (2) the pressure in millibars (mbar) administered and displayed on the equipment screen by the CEB internal sensor after the shot, which corresponded with the pulse or output values. The esthesiometer was designed considering the distance between the air outlet of the equipment and the cornea, as well as the diameter of the air outlet tube. With this design, it can provide the same pressure on the eye as at the instrument’s exit. The distance between the air outlet of the equipment and the ocular surface, as well as the diameter of the air outlet tube, were designed to maintain the same exit and arrival pressures. In a previous study of our research team [21], the correspondences were established as follows: Level 1 = 1–2 mbar; Level 2 = 3–4 mbar; Level 3 = 4–5 mbar; Level 4 = 6–7 mbar; and Level 5 = 8–9 mbar.

The evaluation of corneal sensitivity for each subject was performed separately by two different investigators/ophthalmologists. The first, third, and fourth esthesiometries were performed by investigator 1, and the second by investigator 2. The last esthesiometry was performed after three minutes of topical ocular anesthesia with two eyedrops of double anesthetic solution at 1 mg/mL + 4 mg/mL (tetracaine hydrochloride/oxybuprocaine hydrochloride). Topical ocular anesthesia was used to minimize the influence of the eyelid due to the air jet diffusion.

For all measurements, the esthesiometer was placed in a slit lamp, and pulses of air were applied over the surface of the central part of the volunteer’s cornea. The stimulation threshold was determined using the ascending method of limits, starting with the lowest stimulus level. The pressure value was increased, at which the volunteer verbally communicated that they noticed the air pulse, and positive responses were recorded. The pressure values selected on the instrument and the actual pressure values administered were analyzed to ensure the safety of the procedure.

### 2.6. Safety Assessments

The investigator confirmed and recorded the safety of the CEB after each esthesiometry, as demonstrated by the absence of corneal staining, evaluated with a slit lamp.

### 2.7. Sample Size

The strategy for determining the number of participants in this study involved recruiting as many healthy volunteers as possible to obtain the most robust results. Since this was a pilot study and subjects were included based on availability, the statistical power was calculated post hoc using the G*Power software (version 3.1.9.7) [26]. The pilot study started when 20 volunteers were recruited per center, resulting in a minimum of 80 subjects and a maximum of 200 across all centers.

### 2.8. Data Processing and Statistical Analysis

Measurements obtained from CEB were taken on the slit lamp according to the manufacturer’s instructions. The data were recorded and analyzed individually. Quantitative variables were described as mean and standard deviation (SD), while categorical variables were described as frequency or percentage. The statistical analysis was performed using NCSS software (Kaysville, UT, USA). No covariate was selected for adjustment in the statistical analyses. As the Kolmogorov–Smirnov test rejected the normality of the data, the Wilcoxon non-parametric test was used to compare the values obtained using the esthesiometer by the same researcher in the same eye at different times (intra-observer, measurements 1 and 3) and to compare the level values obtained by two observers in the same eye (inter-observer, measurements 2 and 3). Bland–Altman plots were used to assess the agreement between the two technicians for each study center. All graphs and statistical plots were generated using GraphPad Prism software (version 8.0.2, GraphPad Software Inc., San Diego, CA, USA).

## 3. Results

### 3.1. Participant Characteristics

Ninety-six volunteers were assessed for eligibility, and ninety-one were finally included in the study, with a mean age of 25 (SD 3.46, range 18–30); fifty-eight (63.7%) were female. There were no dropouts or missing data during the study. By centers: Center A recruited twenty-three (25.3%) volunteers with a mean age of 25.35 (SD 2.43, range 18–29), and seventeen were women (73.91%); center B recruited twenty (20.9%) volunteers with a mean age of 25.4 (SD 3.1, range 18–30), and thirteen (65.0%) were women; center C recruited nineteen (22.0%) volunteers with a mean age of 23.5 (SD 4.44, range 18–29), and eleven (57.89%) were women; center D recruited twenty-nine (31.9%) volunteers with a mean age of 24.41 (SD 3.69, range 19–30), and fifteen (51.72%) were women.

Regarding OSDI value, patients at center A have a mean value of 5.53 (SD 7.41), patients at center B have a mean value of 5.10 (SD 4.70), patients at center C have a mean value of 5.32 (SD 4.70), and patients at center D have a mean value of 3.99 (SD 4.22).

### 3.2. Primary Efficacy Endpoint

**Figure 3:**
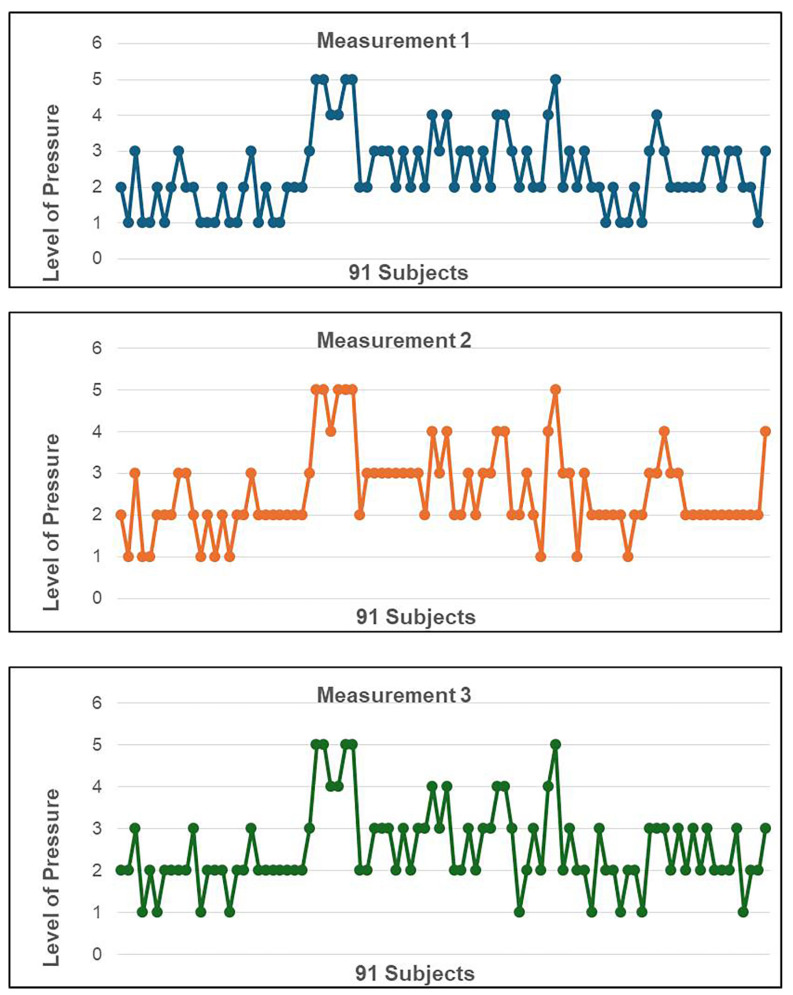
Level of pressure values in the randomized eye after measurements 1, 2, and 3 with the CEB.

In measurements 1, 2, and 3, the mean pressure level at which the air jet was delivered to obtain a response was 2.42 (SD 1.07, range 1–5) in 90 patients tested (Figure 3). The corneal sensitivity reference levels in young, healthy volunteers ranged from Level 1 (13% of corneas assessed), Level 2 (45% of corneas assessed), Level 3 (28% of corneas assessed), Level 4 (9% of corneas assessed), and Level 5 (6% of corneas assessed), and the correspondence pressure in millibars, administered and displayed on the equipment screen by the CEB internal sensor after each shot for these levels, ranged from 3 to 4 mbar for the Level 2, and 4–5 mbar for the Level 3.

Based on the global intraclass correlation coefficient (ICC) of 0.876, calculated from 90 subjects using single measurements per rater, and setting the minimum acceptable ICC under the null hypothesis at 0.50, the achieved statistical power was greater than 99.9% (α = 0.05, one-tailed).

### 3.3. Secondary Efficacy Endpoints

**Figure 4:**
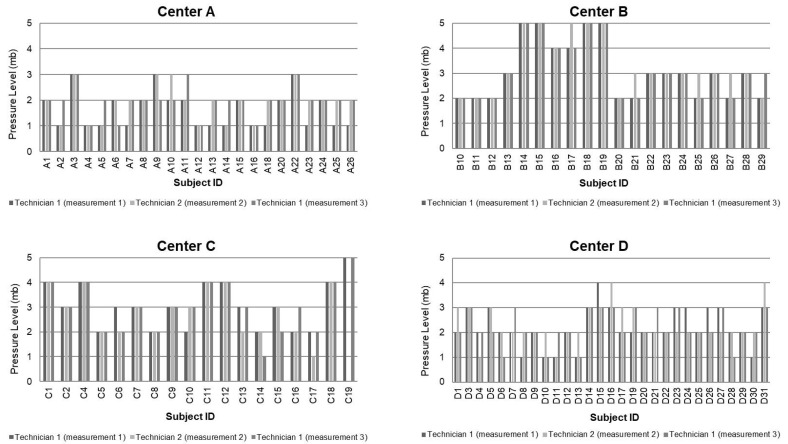
Pressure levels (in millibars) of measurements 1, 2, and 3 with the CEB per center in the randomized eye.

Regarding the reproducibility of the CEB measurements in healthy subjects (measurements 1, 2, and 3), the mean pressure level for each patient was equal, except for some punctual measurements without statistically significant differences (Figure 4). When comparing the intra-observer measurements (measurements 1 and 3) and the inter-observer measurements (measurements 2 and 3) on the same subject without anesthesia, the differences were not statistically significant (Table 1).

**Figure 5:**
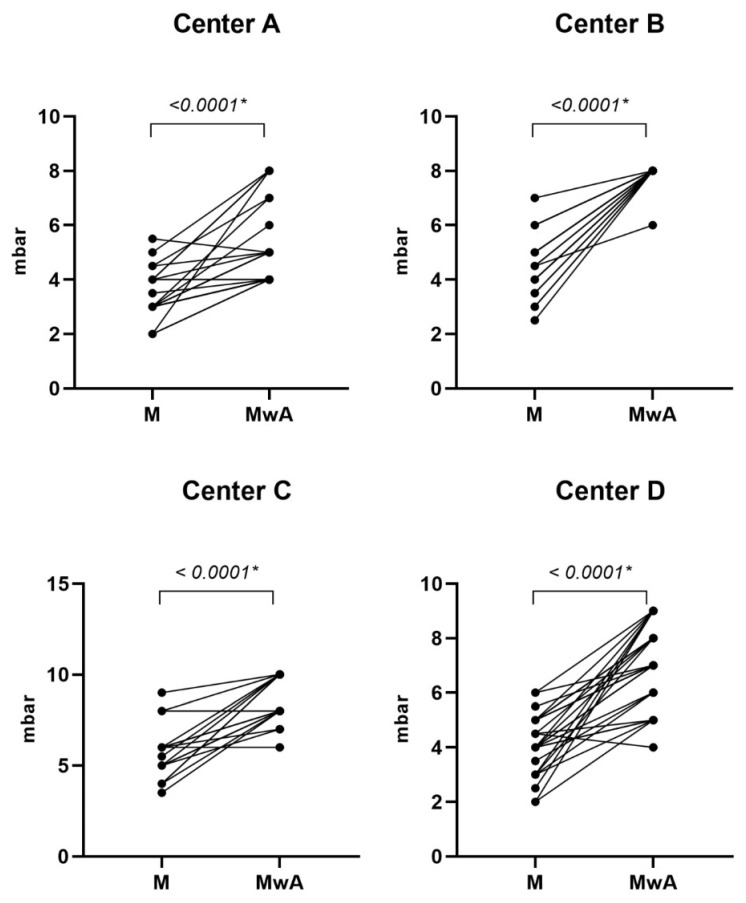
Paired comparisons of corneal sensitivity (in mbar) before (M) and after topical anesthesia (MwA) as measured by Technician 1 in each study center. Each line represents a single subject. A significant increase in threshold was observed after anesthesia in all centers (*p* < 0.0001, Wilcoxon signed-rank test). (*) Statistically significant difference.

**Figure 6:**
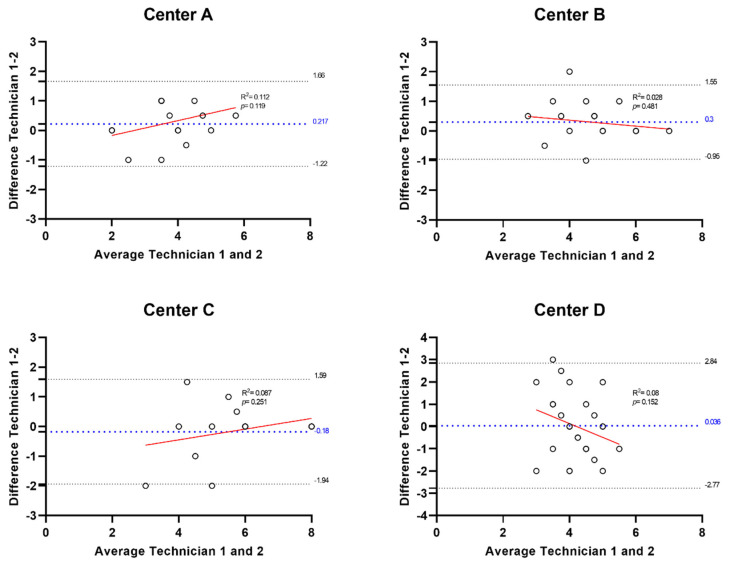
Bland–Altman plots comparing measurements by Technician 1 and Technician 2 in each center.

Regarding the sensitivity of CEB, corneal pressure in 90 healthy volunteers before topical ocular anesthesia had a mean value of 2 (SD 1). After anesthesia, the mean value was 4 (SD 1), with a mean difference of −2 (SD 0) between the two measurements, *p* < 0.0001. The results showed that the air pressure administered to make the cornea sensitive increased after the anesthetic was administered (Figure 5). However, most subjects who have trigger sensitivity at Level 2 or even Level 1 do not notice the delivered air. Fourteen (15.4%) participants from centers A, C, and D did not experience a change in the corneal sensitivity after the ocular anesthesia (A3, A6, A8, A14, A15, A16, A18, A26, D17, D20, D21, D25, D26, and C13) (Supplemental Table S1), and thirteen (14.3%) participants from center B and C showed low sensitivity to the air provided by the esthesiometer and no differences in sensitivity after the ocular anesthesia (B14, B15, B16, B17, B18, B19, C1, C3, C4, C11, C12, C18, and C19) (Supplemental Table S1). One volunteer (C3) did not detect the air released by the esthesiometer in either of the two eyes. The researcher conducted an off-protocol measurement in the patient’s other eye to rule out the possibility that the issue was due to a problem with the air pressure received, an effect of the anesthesia, and to verify that the patient had very low sensitivity, as was finally confirmed. The data from this patient were not included in the data analysis because he did not detect any of the five levels. Supplemental Tables S2–S5 include the values of all measures carried out during the study by centers. Bland–Altman plots comparing the differences between the two technicians for each study center showed that differences were not significant (Figure 6).

### 3.4. Safety Data

No adverse events occurred in this study. No damage was caused to the participant or the user.

## 4. Discussion

According to the data obtained in this study, the CEB could detect a loss of sensitivity with a range of corneal sensitivity pressures in young volunteers, specifically between Level 2 (3–4 mbar) and Level 3 (4–5 mbar). The CEB yielded consistent and reproducible measurements, supporting objective use independent of the operator and demonstrating no significant inter- or intra-observer variability. Therefore, it is confirmed that the use of the esthesiometer does not depend on the technician; rather, it is an objective measure of sensation. When an eye is anesthetized, it loses its sensitivity to light. According to the results obtained, the CEB was able to detect this loss of sensitivity. The sample size was sufficient to detect a high degree of concordance between raters using the CEB. The assessment of corneal sensitivity is of great clinical relevance, especially in patients with dry eye [4,27,28,29]. The comparison of measurements with the nylon filament in the case of the CBE is complex, as other authors have found in previous studies [23]. In the previous study, the CEB was effective and easy to use for evaluating corneal sensitivity, with a repeatability SD of 0 in 96.3% of the measurements of the eyes with pathology, and healthy eyes were sensitive to lower pressure levels [21]. A study was conducted in healthy volunteers comparing CEB vs. CBE [30] where the investigators concluded that the CEB is a safe, reliable, and user-independent tool with advantages over the CBE. The strengths of the CEB are its non-contact use, reduced patient apprehension, and a wider stimulus range. However, as sensitivity thresholds differ, the two devices cannot be used interchangeably.

The CEB was safe, and no adverse effects or damage to the corneal surface were found, because the CEB is a non-contact device applied at a fixed distance to the subject and cannot cause corneal erosions. Furthermore, its design allows operator-independent reproducibility. These two aspects represent a significant advantage over the Cochet-Bonnet esthesiometer, considered the gold standard. Before the first study was published [21], an internal study was conducted with nineteen healthy volunteers, with a mean age of 23.50 years (SD 3.62), to assess the safety and repeatability of the CEB versus the CBE. This study did not reveal significant differences between the two devices.

In 1996, Murphy et al. conducted a study using an esthesiometer with air pulses, with normality values of 0.342 ± 0.068 millibars [31]. In 1998, the same author conducted a study in healthy patients comparing the air esthesiometer with the CBE [22]. They performed analyses using different air jet diameters (0.3, 0.9, 1, and 2 mm) and stimulus durations (0.5, 0.9, and 1.5 s) to compare the two devices. The results demonstrated that the CBE had severe design deficiencies, which limited its ability to accurately measure corneal sensitivity at low stimulus thresholds.

Other studies have been conducted using the Belmonte esthesiometer. This non-contact esthesiometer applies pneumatic stimuli, a mixture of air and 98.5% CO_2_, over the center of the corneal surface via an electronic valve [18]. Although not commercially available, standard and modified versions of this device have been used in several studies in patients with healthy and pathological eyes [32,33,34]. A study revealed that a non-contact esthesiometer allowed superior reproducibility of the stimulus and better control over stimulus characteristics [35].

### Study Limitations

One limitation of the study is that the included patients do not represent all age groups. Consequently, the reference values obtained are only applicable to the young population, especially those between 18 and 20 years old, as their corneas are healthier. Further research is needed to include a broader age range and encompass conditions known to affect corneal sensitivity, such as herpetic keratitis, diabetes mellitus, and surgical procedures like LASIK [36,37,38,39,40].

Another potential limitation is the observed differences between centers, which may be attributed to variations in the interpretation of the study protocol, including the precise position of the alignment of the two light sources at the edge of the pupil, or subjects that did not change or had low baseline sensitivity (15–14%) maybe due to anatomical variations. However, adherence to the protocol was confirmed during the monitoring visits, and the observed differences were limited to a small number of patients. Additionally, comparative studies involving the CBE are recommended to assess the equivalence or potential superiority of the device under investigation.

## 5. Conclusions

The CEB yielded consistent and reproducible measurements in young volunteers with healthy eyes, enabling objective, observer-independent assessment and facilitating the detection of significant loss of sensitivity.

## Figures and Tables

**Table 1:** Intra- and inter-observer statistical analysis, Wilcoxon Test.

	Test. Intra-Observer		Test. Inter-Observer	
	Normal Approximation	Normal Approximation with C.C.	Normal Approximation	Normal Approximation with C.C.
**Z value**	1.4606	1.4582	0.7071	0.7047
***p*-value**	0.14413	0.14479	0.4795	0.48098

## Data Availability

The data that support the findings of this study are available from the corresponding author upon reasonable request.

## Supplementary Materials

The following supporting information can be downloaded at: https://www.mdpi.com/article/10.3390/diagnostics15172208/s1, Table S1. Inclusion tests results by center (A, center A; B, center B; C, center C; D, center D). Table S2. Values of all measures carried out during the study in center A. Table S3. Values of all measures carried out during the study in center B. Table S4. Values of all measures carried out during the study in center C. Table S5. Values of all measures carried out during the study in center D.

## Abbreviations

The following abbreviations are used in this manuscript:

CBE	Cochet-Bonnet esthesiometer
CEB	Corneal Esthesiometer Brill
FDA	Food and Drug Administration
LEDs	Light-emitting diodes
OSDI	Ocular surface disease index
SD	Standard deviation
