# Supplementary material for: Functioning and Safety of the Non-Invasive Corneal Esthesiometer Brill: A Multicenter Study
Source: Diagnostics (Basel). 2025 Aug 30;15(17):2208. doi: 10.3390/diagnostics15172208 (PMC12427739; doi:10.3390/diagnostics15172208)
Supplement: Supplementary file 1 [file diagnostics-15-02208-s001.zip › diagnostics-3823232-supplementary.pdf]

Title: Functioning and Safety of the Non-Invasive Corneal Esthesiometer Brill: A Multicenter Study

Supplemental Tables

Table S1. Inclusion tests results by center (A, center A; B, center B; C, center C; D, center D).

Subject	Age	Gender	OSDI value	SLB	Change in the corneal sensitivity after topical anesthesia (measurement 4)
A1	27	M	6,25	OK	Yes
A2	28	F	4,16	OK	Yes
A3	25	M	0,00	OK	No
A4	25	F	0,00	OK	Yes
A5	29	M	0,00	OK	Yes
A6	28	F	2,08	OK	No
A7	30	M	0,00	OK	Yes
A8	25	F	0,00	OK	No
A9	23	F	0,00	-	Yes
A10	22	F	2,08	OK	Yes
A11	28	F	0,00	OK	Yes
A12	26	F	2,08	OK	Yes
A13	25	F	4,16	OK	Yes
A14	22	F	4,16	OK	No
A15	24	F	4,16	OK	No
A16	26	F	4,16	OK	No
A17	28	F	14,58	OK	Yes
A18	23	F	0,00	OK	No
A19	27	F	18,75	OK	Yes
A20	25	F	4,16	OK	Yes
A21	25	F	33,33	OK	Yes
A22	28	F	8,33	OK	Yes
A23	24	M	8,33	OK	Yes
A24	22	F	10,41	OK	Yes
A25	22	M	8,33	OK	Yes
A26	22	F	4,16	OK	No
B10	30	M	2,08	OK	Yes
B11	18	F	0	OK	Yes
B12	23	F	4,16	OK	Yes
B13	24	F	0	OK	Yes
B14	21	F	0	OK	Low sensitivity, no differences
B15	28	M	0	OK	Low sensitivity, no differences
B16	27	M	6,25	OK	Low sensitivity, no differences
B17	28	F	12,5	OK	Low sensitivity, no differences
B18	27	F	0	OK	Low sensitivity, no differences
B19	27	F	0	OK	Low sensitivity, no differences
B20	25	F	10,41	OK	Yes
B21	24	F	10,41	OK	Yes
B22	28	F	8,33	OK	Yes
B23	26	M	8,33	OK	Yes

Subject	Age	Gender	OSDI value	SLB	Change in the corneal sensitivity after topical anesthesia (measurement 4)
B24	26	M	0	OK	Yes
B25	24	M	10,41	OK	Yes
B26	30	F	2,08	OK	Yes
B27	22	M	10,41	OK	Yes
B28	22	F	6,25	OK	Yes
B29	28	F	10,41	OK	Yes
C1	24	F	8,33	OK	Low sensitivity, no differences
C2	28	F	12,5	OK	Yes
C3	25	M	0	OK	Low sensitivity, no differences*
C4	29	F	2,08	OK	Low sensitivity, no differences
C5	24	M	0	OK	Yes
C6	18	F	6,25	OK	Yes
C7	28	M	0	OK	Yes
C8	29	F	0	OK	Yes
C9	29	F	2,08	OK	Yes
C10	20	M	12,5	OK	Yes
C11	19	M	0	OK	Low sensitivity, no differences
C12	19	F	4,16	OK	Low sensitivity, no differences
C13	20	F	10,4	OK	No
C14	20	F	12,5	OK	Yes
C15	29	M	4,16	OK	Yes
C16	20	M	0	OK	Yes
C17	28	F	4,16	OK	Yes
C18	20	M	8,33	OK	Low sensitivity, no differences
C19	19	F	8,33	OK	Low sensitivity, no differences
D1	22	M	4,16	OK	Yes
D2	0	F	14,53	NOK	Yes
D3	27	M	12,5	OK	Yes
D4	29	F	4,16	OK	Yes
D5	26	M	0	OK	Yes
D6	27	F	2,03	OK	Yes
D7	26	F	0	OK	Yes
D8	22	F	5	OK	Yes
D9	20	F	10,42	OK	Yes
D10	21	M	0	OK	Yes
D11	20	F	4,54	OK	Yes
D12	22	F	2,27	OK	Yes
D13	27	F	8,33	OK	Yes
D14	18	M	8,33	OK	Yes
D15	18	M	4,16	OK	Yes
D16	22	M	2,08	OK	Yes
D17	22	M	0	OK	No
D18	22	F	22,92	NOK	Yes
D19	28	F	12,5	OK	Yes
D20	20	F	12,5	OK	No
D21	20	F	6,25	OK	No
D22	25	F	0	OK	Yes

Subject	Age	Gender	OSDI value	SLB	Change in the corneal sensitivity after topical anesthesia (measurement 4)
D23	26	F	8,33	OK	Yes
D24	27	M	0	OK	Yes
D25	28	F	0	OK	No
D26	22	F	6,25	OK	No
D27	28	M	0	OK	Yes
D28	27	F	0	OK	Yes
D29	29	M	2,08	OK	Yes
D30	29	F	4,16	OK	Yes
D31	30	M	0	OK	Yes

*The patient did not detect the air released by the aesthesiometer in either of the two eyes.

Abbreviations: M, male; F, female; OSDI, Ocular surface disease index; SLB, slit-lamp biomicroscopy.

Table S2. Values of all measures carried out during the study in center A.

Subject ID	Level of pressure detected with *Corneal Esthesiometer BRILL*					Pressure values (mbar)				
	Technician 1 (measurement 1)	Technician 2 (measurement 2)	Technician 1 (measurement 3)	Technician 1 (measurement 4, with anesthesia)	Technician 1 (measurement eye not tested)	Technician 1 (measurement 1)	Technician 2 (measurement 2)	Technician 1 (measurement 3)	Technician 1 (measurement 4, with anesthesia)	Technician 1 (measurement eye not tested)
A1	2	2	2	3	2	4	4	4	5	4
A2	1	1	2	4	1	2	2	4	7	2
A3	3	3	3	3	2	6	6	5	5	4
A4	1	1	1	2	1	2	2	2	4	2
A5	1	1	2	2	1	2	2	4	4	2
A6	2	2	1	2	1	4	4	2	4	2
A7	1	2	2	4	1	2	4	4	6	2
A8	2	2	2	2	2	3	4	4	4	4
A9	3	3	2	4	3	5	5	4	7	5
A10	2	3	2	5	2	4	5	4	8	4
A11	2	2	3	3	3	4	4	5	5	5
A12	1	1	1	5	1	2	2	2	8	2
A13	1	2	2	3	2	2	4	4	5	4
A14	1	1	2	2	1	2	2	4	4	2
A15	2	2	2	2	1	4	3	4	4	2
A16	1	1	1	2	2	2	2	2	4	4
A18	1	2	2	2	2	2	4	4	4	4
A20	2	2	2	5	3	4	4	4	8	6
A22	3	3	3	4	3	5	5	5	8	5
A23	1	2	2	3	1	2	4	4	5	2
A24	2	2	2	2	2	4	4	4	4	2
A25	1	2	2	3	1	2	4	4	5	2
A26	1	2	2	2	1	2	4	4	4	2

Table S3. Values of all measures carried out during the study in center B.

Subject ID	Level of pressure detected with *Corneal Esthesiometer BRILL*					Pressure values (mbar)				
	Technician 1 (measurement 1)	Technician 2 (measurement 2)	Technician 1 (measurement 3)	Technician 1 (measurement 4, with anesthesia)	Technician 1 (measurement eye not tested)	Technician 1 (measurement 1)	Technician 2 (measurement 2)	Technician 1 (measurement 3)	Technician 1 (measurement 4, with anesthesia)	Technician 1 (measurment eye not tested)
B10	2	2	2	*>5*	2	4	4	4	*out of range*	4
B11	2	2	2	*>5*	2	4	4	4	*out of range*	4
B12	2	2	2	*>5*	2	3	4	4	*out of range*	3
B13	3	3	3	*>5*	3	5	5	5	*out of range*	5
B14	5	5	5	*>5*	5	6	6	6	*out of range*	6
B15	5	5	5	*>5*	5	7	7	7	*out of range*	7
B16	4	4	4	*>5*	4	5	5	5	*out of range*	5
B17	4	5	4	*>5*	4	5	6	5	*out of range*	5
B18	5	5	5	*>5*	5	6	6	6	*out of range*	6
B19	5	5	5	*>5*	5	6	6	6	*out of range*	6
B20	2	2	2	*>5*	2	3	3	2	*out of range*	3
B21	2	3	2	*>5*	3	4	5	4	*out of range*	5
B22	3	3	3	*>5*	3	5	4	5	*out of range*	5
B23	3	3	3	*>5*	3	5	5	4	*out of range*	5
B24	3	3	3	*>5*	3	5	5	4	*out of range*	5
B25	2	3	2	5	3	3	5	3	6	5
B26	3	3	3	*>5*	3	5	5	4	*out of range*	4
B27	2	3	2	*>5*	2	3	4	3	*out of range*	3
B28	3	3	3	*>5*	3	4	4	4	*out of range*	4
B29	2	2	3	*>5*	3	3	3	4	*out of range*	4

>5: no more higher levels available to test.

out of range: no pressure was obtained with the esthesiometer.

Table S4. Values of all measures carried out during the study in center C.

Subject ID	Level of pressure detected with *Corneal Esthesiometer BRILL*					Pressure values (mbar)				
	Technician 1 (measurement 1)	Technician 2 (measurement 2)	Technician 1 (measurement 3)	Technician 1 (measurement 4, with anesthesia)	Technician 1 (measurement eye not tested)	Technician 1 (measurement 1)	Technician 2 (measurement 2)	Technician 1 (measurement 3)	Technician 1 (measurement 4, with anesthesia)	Technician 1 (measurement eye not tested)
C1	4	4	4	5	4	6	6	6	7	6
C2	3	3	3	5	2	5	5	5	7	3
C4	4	4	4	5	4	6	6	6	8	6
C5	2	2	2	5	3	4	4	4	10	6
C6	3	2	2	4	2	6	4	4	8	4
C7	3	3	3	4	3	6	6	6	8	6
C8	2	2	2	5	2	4	4	4	10	4
C9	3	3	3	5	3	6	6	6	10	6
C10	2	3	3	5	4	5	6	6	10	8
C11	4	4	4	4	4	8	8	8	8	8
C12	4	4	4	4	4	8	8	8	8	8
C13	3	2	3	3	3	6	4	6	6	6
C14	2	2	1	4	2	5	5	2	8	5
C15	3	3	2	4	3	6	6	4	8	6
C16	2	2	3	5	2	4	4	6	10	4
C17	2	1	2	4	3	4	2	4	8	6
C18	4	4	4	*>5*	4	8	8	8	*out of range*	8
C19	5	*>5*	5	*>5*	4	9	*out of range*	9	*out of range*	7

>5: no more higher levels available to test.

out of range: no pressure was obtained with the esthesiometer.

Table S5. Values of all measures carried out during the study in center D.

Subject ID	Level of pressure detected with *Corneal Esthesiometer BRILL*					Pressure values (mbar)				
	Technician 1 (measurement 1)	Technician 2 (measurement 2)	Technician 1 (measurement 3)	Technician 1 (measurement 4, with anesthesia)	Technician 1 (measurement eye not tested)	Technician 1 (measurement 1)	Technician 2 (measurement 2)	Technician 1 (measurement 3)	Technician 1 (measurement 4, with anesthesia)	Technician 1 (measurement eye not tested)
D1	2	3	2	*>5*	2	4	5	4	*out of range*	4
D3	3	3	3	5	3	5	5	5	8	5
D4	2	1	2	3	1	3	2	3	5	2
D5	3	3	2	5	3	5	5	3	8	5
D6	2	2	1	5	2	3	3	2	9	3
D7	2	2	3	5	2	4	5	6	8	4
D8	1	2	2	*>5*	2	1	3	3	*out of range*	3
D9	2	2	2	4	2	5	4	5	8	5
D10	1	2	1	3	2	3	4	3	6	4
D11	1	1	2	3	1	3	3	4	6	3
D12	2	2	2	4	1	4	4	4	7	3
D13	1	2	1	3	1	2	4	2	5	2
D14	3	3	3	4	3	5	5	5	7	5
D15	4	3	3	4	4	7	5	5	7	7
D16	3	4	3	*>5*	3	5	6	5	*out of range*	5
D17	2	3	2	3	2	4	5	4	6	4
D19	2	3	3	5	2	4	5	5	8	4
D20	2	2	2	3	2	4	4	4	5	4
D21	2	2	3	3	3	4	4	5	5	5
D22	2	2	2	4	2	4	4	4	7	4
D23	3	2	3	4	3	6	4	5	7	5
D24	3	2	2	4	3	6	4	4	7	5
D25	2	2	2	3	3	4	4	4	5	5
D26	3	2	2	2	2	5	4	4	4	4

Subject ID	Level of pressure detected with *Corneal Esthesiometer BRILL*					Pressure values (mbar)				
	Technician 1 (measurement 1)	Technician 2 (measurement 2)	Technician 1 (measurement 3)	Technician 1 (measurement 4, with anesthesia)	Technician 1 (measurement eye not tested)	Technician 1 (measurement 1)	Technician 2 (measurement 2)	Technician 1 (measurement 3)	Technician 1 (measurement 4, with anesthesia)	Technician 1 (measurement eye not tested)
D27	3	2	3	4	2	5	4	5	7	4
D28	2	2	1	5	2	4	4	2	9	4
D29	2	2	2	*>5*	3	4	4	4	*out of range*	5
D30	1	2	2	4	2	2	4	4	8	4
D31	3	4	3	5	3	6	7	6	9	6

>5: no more higher levels available to test.

out of range: no pressure was obtained with the esthesiometer.
